# Geological and climatic changes in quaternary shaped the evolutionary history of *Calibrachoa heterophylla*, an endemic South-Atlantic species of petunia

**DOI:** 10.1186/1471-2148-13-178

**Published:** 2013-08-29

**Authors:** Geraldo Mäder, Jéferson N Fregonezi, Aline P Lorenz-Lemke, Sandro L Bonatto, Loreta B Freitas

**Affiliations:** 1 Laboratory of Molecular Evolution - Department of Genetics, Universidade Federal do Rio Grande do Sul, Porto Alegre, RS, Brazil; 2 Laboratory of Genomic and Molecular Biology, Pontifícia Universidade Católica do Rio Grande do Sul, Porto Alegre, RS, Brazil

**Keywords:** South-Atlantic coastal plain, Phylogeography, Pleistocene, Quaternary, Genetic diversity, Climatic changes, Petunia

## Abstract

**Background:**

The glacial and interglacial cycles that characterized the Quaternary greatly affected the distribution and genetic diversity of plants. In the Neotropics, few phylogeographic studies have focused on coastal species outside of the Atlantic Rainforest. Climatic and sea level changes during the Quaternary played an important role in the evolutionary history of many organisms found in coastal regions. To contribute to a better understanding of plant evolution in this environment in Southern South America, we focused on *Calibrachoa heterophylla* (Solanaceae), an endemic and vulnerable wild petunia species from the South Atlantic Coastal Plain (SACP).

**Results:**

We assessed DNA sequences from two cpDNA intergenic spacers and analyzed them using a phylogeographic approach. The present phylogeographic study reveals the influence of complex geologic and climatic events on patterns of genetic diversification. The results indicate that *C. heterophylla* originated inland and subsequently colonized the SACP; the data show that the inland haplogroup is more ancient than the coastal one and that the inland was not affected by sea level changes in the Quaternary. The major diversification of *C*. *heterophylla* that occurred after 0.4 Myr was linked to sea level oscillations in the Quaternary, and any diversification that occurred before this time was obscured by marine transgressions that occurred before the coastal sand barrier’s formation. Results of the Bayesian skyline plot showed a recent population expansion detected in *C. heterophylla* seems to be related to an increase in temperature and humidity that occurred at the beginning of the Holocene.

**Conclusions:**

The geographic clades have been formed when the coastal plain was deeply dissected by paleochannels and these correlate very well with the distributional limits of the clades. The four major sea transgressions formed a series of four sand barriers parallel to the coast that progressively increased the availability of coastal areas after the regressions and that may have promoted the geographic structuring of genetic diversity observed today. The recent population expansion for the entire species may be linked with the event of marine regression after the most recent sea transgression at ~5 kya.

## Background

The glacial and interglacial cycles that characterized the Pleistocene had major effects on the distribution and genetic diversity of plant species in the Northern Hemisphere caused by the advance and retreat of large ice sheets at higher latitudes [1,2]. In South America, fully formed glaciers occurred only in the Andes, and the main climatic effects on most of the region were that closed vegetation types (forests) alternated with open formations (grasslands) between the glacial and interglacial periods [3]. This might have resulted in more complex histories regarding the establishment and composition of current vegetation than simple extinctions of local biota and the formation of typical glacial refugia. In the Neotropics, most of the phylogeographic studies have focused on eastern South America [4,5] and have investigated continental species from the Atlantic Rainforest e.g., [6], although a few have analyzed species from open areas such the grasslands located in the highlands and in the Pampas region of southern South America [7,8].

After the Last Glacial Maximum (LGM) and at the beginning of the Holocene, approximately 11,700 years ago [9], the climate began to change. Evidence indicates a strong environmental change associated with the expansion of vegetation in southern South America due to a climate markedly warmer and moister than in the Pleistocene [10-12]. The accentuated moisture in this phase was also reported from the South Atlantic Coastal Plain (SACP) and adjacent area [13,14]. According to these references, the pollen concentration of individual taxa increased dramatically at the beginning of the Holocene, providing evidence for rapid climatic change.

Several studies [5-8,15,16] have shown that demographic changes in South American plants are related to Quaternary climatic oscillations. Moreover, changes in sea level due to melting glaciers in the interglacial periods played an important role in the evolutionary history of many coastal region inhabitants [17]. Studies of South American coastal regions are rare despite the potentially interesting evolutionary history of species in these areas, which have been strongly influenced by changes in sea level during glacial and interglacial periods [8]. However, in the Northern Hemisphere, studies of coastal plants have been conducted more often e.g., [18,19] and have found that changes in habitat availability and Quaternary sea level changes were responsible for the genetic structure of Asteraceae species in the Mediterranean Basin. Profound effects of Quaternary climatic cycling, tectonic shifts and changes in river dynamics, as well as sea level fluctuations, were highlighted as playing a role in shaping the genetic structure and phylogeographic patterns of plants [20].

**Figure 1:**
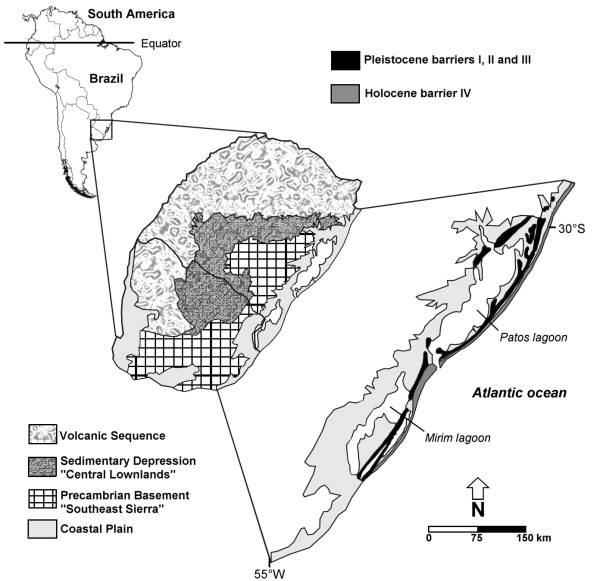
**South Atlantic coastal plain map.** Southern Brazil and Uruguay physiographic map modified from Weschenfelder et al*.*[40], indicating South Atlantic Coastal Plain location and Barrier–Lagoon Systems I to IV positions according Tomazelli & Villwock [21].

The South Atlantic Coastal Plain is the largest coastal plain in South America, covering approximately 33,000 Km^2^, from 28° north to 34° south. It is a flat area of lowlands occupied mostly by large systems of coastal lakes. It extends NE-SW for approximately 600 Km and has an average width of 60–70 Km in the south central portion and 15–20 Km in the northern portion (Figure 1; [21]). The main factors that formed the SACP as it is today were oscillatory glaciation cycles during the Pleistocene, which promoted sea level transgressions and regressions [22]. During the Late Quaternary, four sand barriers parallel to the coast were formed and identified in the SACP; three of these barriers originated during the Pleistocene, and one originated during the Holocene. They were associated with the limits of the marine transgressions of the four major interglacial periods in the last 1 Myr (million years) that occurred in the marine isotope stages (MIS) 11, 9, 5, and 1, which are referred to as Barrier-Lagoon Systems I to IV, respectively (Figure 1; [21,22]).

After Quaternary sea level changes and the establishment of the current SACP, vegetation from the west could expand into this new environment and adapt to different conditions of salinity, soil composition, climate and hydric regime. Many plant species in the SACP originated in Andean–Pampean areas and invaded the subtropical region during the Pleistocene, especially from colder and higher elevated regions [23-25]. The central lowlands (Sedimentary depression; Figure 1) are considered an important gateway for the colonization of the SACP by Andean taxa. Small patches or long strips of gallery forests occur in the central lowlands along rivers between extensive areas of grassy vegetation [26]. This mosaic of plant species was also influenced by the complex history and geography of the SACP region, which contains dunes, lakes, lagoons, and channels that could represent barriers or corridors to gene flow for many species that currently inhabit the area.

In the present work, we chose *Calibrachoa heterophylla* (Sendtn.) Wijsman (Solanaceae) as a model to study phylogeographic patterns in the SACP and thus better understand plant evolution in coastal environments in South America. *Calibrachoa heterophylla* is an endemic and vulnerable wild petunia species with a distribution range predominantly in the SACP, where it inhabits dunes and sandy grasslands. In addition to being found in the coastal region, this species also inhabits inland regions of the same longitude, including sandy soils that are distant (more than 500 Km) from the South Atlantic Coast. The ancestors of wild petunias have an Andean-Pampean origin, with a recent colonization of the present geographical range coupled with a fast and recent morphological radiation [27] and it was demonstrated [7] that much of the diversification of wild petunias occurred during the Pleistocene. The disjunct distribution and the recent evolutionary history of *C. heterophylla* make it an excellent model for understanding the Quaternary evolutionary processes of plants in the South Atlantic Coast.

Genetic markers can shed light on phylogeographical history of organisms [28]. Chloroplast DNA (cpDNA) sequences are commonly used assess geographic structure in plants [29,30] and they are especially informative in relatives of petunia [7,31-33]. Plastid markers have some merits over nuclear markers for phylogeographical studies. These properties include the absence of recombination, low effective population size and the conservative mutation rate, allowing for primers to be designed over a wide taxonomic range [34,35]. These classical cpDNA markers can answer perfectly well phylogeographic questions minimizing the effect of recent gene flow, which could mask historic biogeographic events [36].

The main aim of this work was to investigate how past climatic and geological changes in the SAPC could have affected the evolutionary history of *C. heterophylla*. Specifically we asked the following questions: (i) Did *C. heterophylla* colonize the SACP from inland populations growing in ancient soils that were not affected by sea level changes, or did the species arise in the SACP and then were brought or migrated inland later? (ii) Did the environmental and sea level changes during the Quaternary result in allopatric processes that isolated and shaped the genetic structure of populations? (iii) Did Holocene climate changes result in changes of population size in *C. heterophylla*? To answer these questions, we assessed the DNA sequences of two cpDNA intergenic spacers (*trnH-psbA* and *trnS-trnG*) that have been successfully employed in population studies of wild petunias [7,31]. To relate the genetic variability of cpDNA markers to the geological and climatic history of the SACP during the Quaternary, several phylogeographic and phylogenetic analyses, including the dating of the formation of major clades, were performed.

## Results

### Genetic diversity

The mean number of individuals genotyped per population was 17.6. The representative lengths of the alignment for *trnH-psbA* and *trnS*-*trnG* were 456 bp and 784 bp, respectively. Overall, 32 sites were informative, 16 in each region, resulting in a value of 0.41 ± 0.22 for nucleotide diversity (*π*%) and a value of 0.879 ± 0.010 for haplotype diversity (*h*). Moreover, 27 different haplotypes were identified from concatenated spacers (Additional file 1). The marker *trnH-psbA* had diversity indices slightly higher than *trnS-trnG*, though the amount of variability is similar (Table 1). However, intra-population diversity varied widely throughout the populations sampled. Five populations were monomorphic and most others exhibited relatively low nucleotide diversity. P6 and P7 populations had the highest values of nucleotide diversity. The summary statistics obtained for the markers and populations are shown in Tables 1 and 2, respectively.

**Table 1:** Summary statistics for the datasets used

***Marker***	***π *(SD) %**	***h *(SD)**	**Length/bp**	**Ts**	**Tv**	**Indels**
*trnH-psbA*	0.46 (0.28)	0.8479 (0.011)	456	4	12	0
*trnS-trnG*	0.38 (0.22)	0.7978 (0.014)	779–784	5	10	1*
Concatenated	0.41 (0.22)	0.8 (0.010)	1240–1236	9	22	1*

*π*, nucleotide diversity; *h*, haplotype diversity; SD, standard deviation;

* Duplication of 5 bp; Tv, transversion; Ts, transition.

**Table 2:** Summary statistics for each population

***Population***	***Haplotypes (n)***	***SAMOVA group***	***Haplogroups***	***h (SD)***	***π (SD)%***
P1	H14(27); H15(4); H16(1); H17(1)	G1	South Coast (green)	0.324 ± 0.098	0.028 ± 0.031
P2	H5(14); H7(2); H8(10)	G2	Central Coast (blue)	0.579 ± 0.057	0.052 ± 0.047
P3	H5 (2)	G2	Central Coast (blue)	0	0
P4	H5(27); H11(1); H12(4), H13(1)	G2	Central Coast (blue)	0.324 ± 0.098	0.033 ± 0.034
P5	H5(12); H6(1)	G2	Central Coast (blue)	0.154 ± 0.126	0.013 ± 0.021
P6	H18(15); H19(11)	G2	Central (blue) and North Coast (red)	0.508 ± 0.040	0.450 ± 0.249
P7	H19(26); H20(1); H21(3)	G3	Central (blue) and North Coast (red)	0.246 ± 0.098	0.156 ± 0.101
P8	H2(20); H9(3); H10(1)	G3	North Coast (red)	0.301 ± 0.112	0.030 ± 0.033
P9	H2(22); H3(1)	G3	North Coast (red)	0.087 ± 0.078	0.007 ± 0.015
P10	H27 (10)	G3	North Coast (red)	0	0
P11	H1 (3)	G4	Mainland (yellow)	0	0
P12	H4(3); H22(6); H23(1); H24(1)	G4	Mainland (yellow)	0.673 ± 0.123	0.070 ± 0.060
P13	H4(7); H25(1); H26(1)	G4	Mainland (yellow)	0.417 ± 0.191	0.067 ± 0.060
P14	H4(4)	G4	Mainland (yellow)	0	0
**Total**	**27**			**0.879 ± 0.010**	**0.409 ± 0.220**

*n*, number of samplings; *h*, haplotype diversity; *π*, nucleotide diversity; SD, standard deviation.

### Phylogeography and population structure

The degree of genetic structure of the populations was estimated by AMOVA, which indicated that 85.1% of the variation was among populations and 14.9% was within populations. SAMOVA analysis was used to infer the best grouping of populations on the basis of molecular variation. The configuration of four groups was considered the best by presenting the highest value of Φ_CT_ (0.691; p < 0.001). Four groups were inferred: a group in the extreme south of the SACP (G1), a group in the central SACP (G2), a group in north of the SACP (G3) and a final group comprising inland populations (G4). Populations in each group are listed in Table 2.

A Mantel test showed a significant and moderate correlation between genetic and geographical distances between the populations (correlation coefficient = 0.39; p < 0.001). Spatial autocorrelation analysis with Alleles in Space (AIS), for the whole species, agrees with the above result, but showed a direct correlation between genetic and geographic distances up to approximately 230 km, after that the genetic divergence is maximum and shows no further increase (data not shown). Since the maximum distances between individuals within the groups are around the above value (~300 km), this suggests that, as expected, this isolation by distance pattern occurs mainly within groups. This is supported by the similar results of the spatial autocorrelation analyses for each of the two clusters with large sample sizes (G2 and G3).

**Figure 2:**
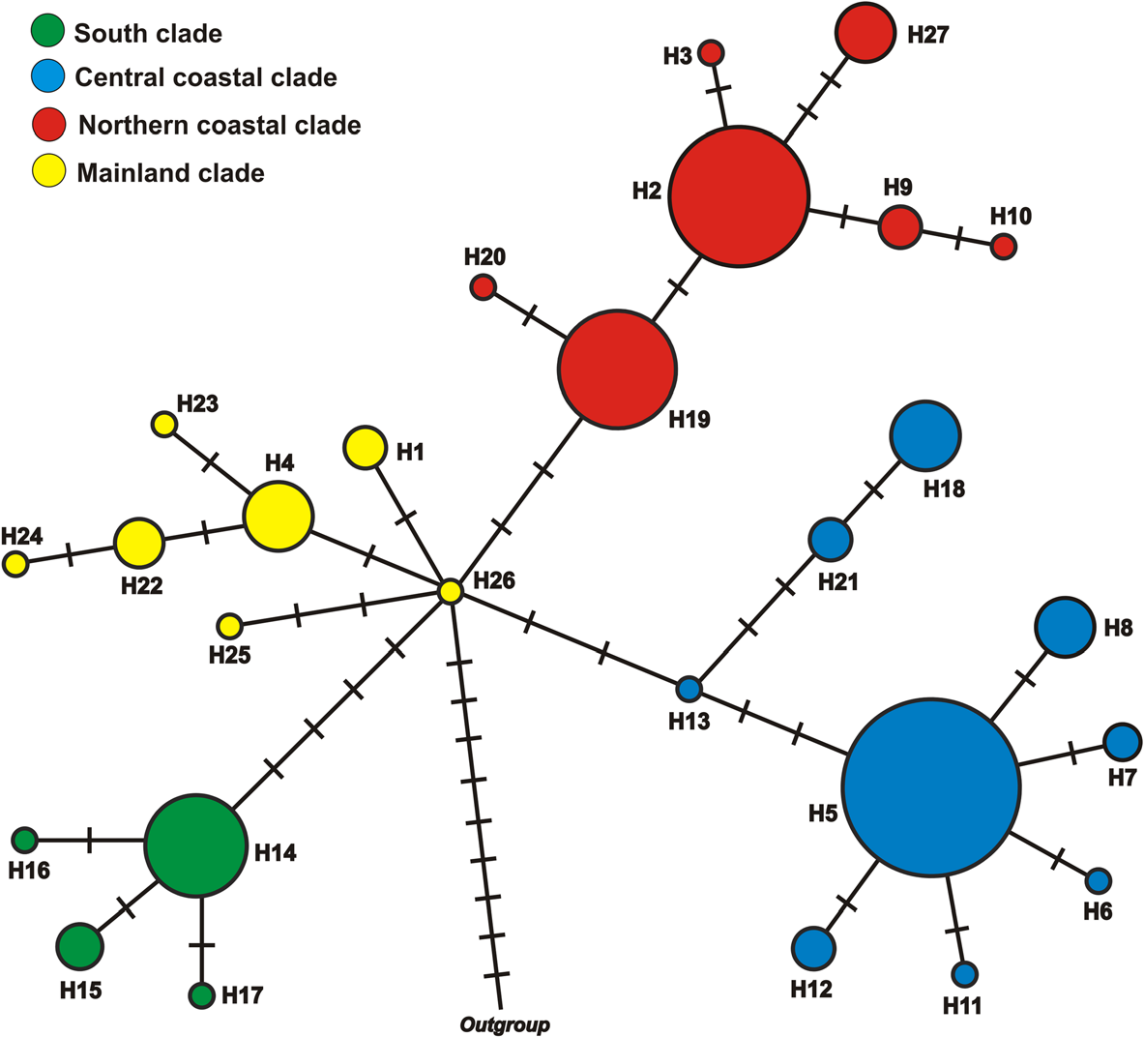
**Haplotypes network.** Evolutionary relationships among haplotypes of *Calibrachoa heterophylla* cpDNA using Median-joining network approach. Colors identified the haplogroups. Circle sizes are proportional to haplotype frequency and crossed lines are substitutions inferred in the branches.

**Figure 3:**
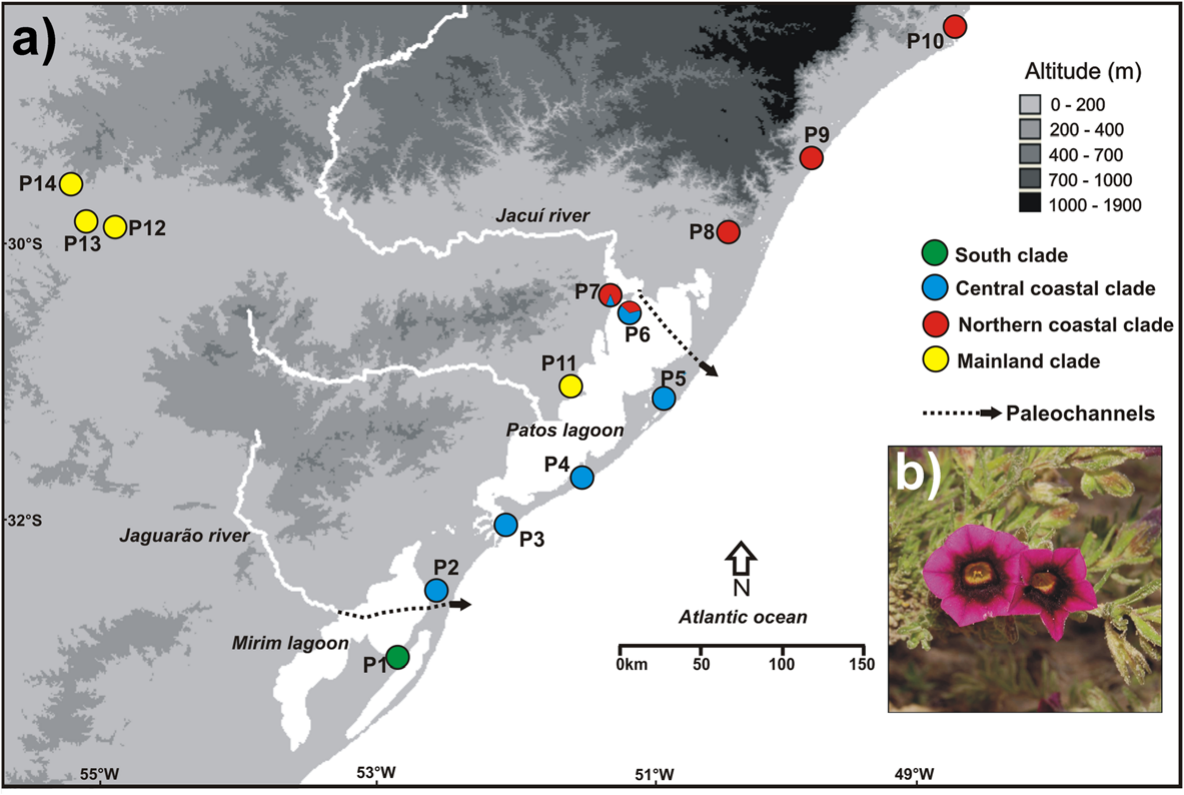
**Collection site location. a)** Map showing the sampling sites. The colors of the populations are related to the haplogroups identified by the network and Bayesian chronogram. Dotted lines represent paleochannels [40,51]. **b)***Calibrachoa heterophylla* flower.

**Figure 4:**
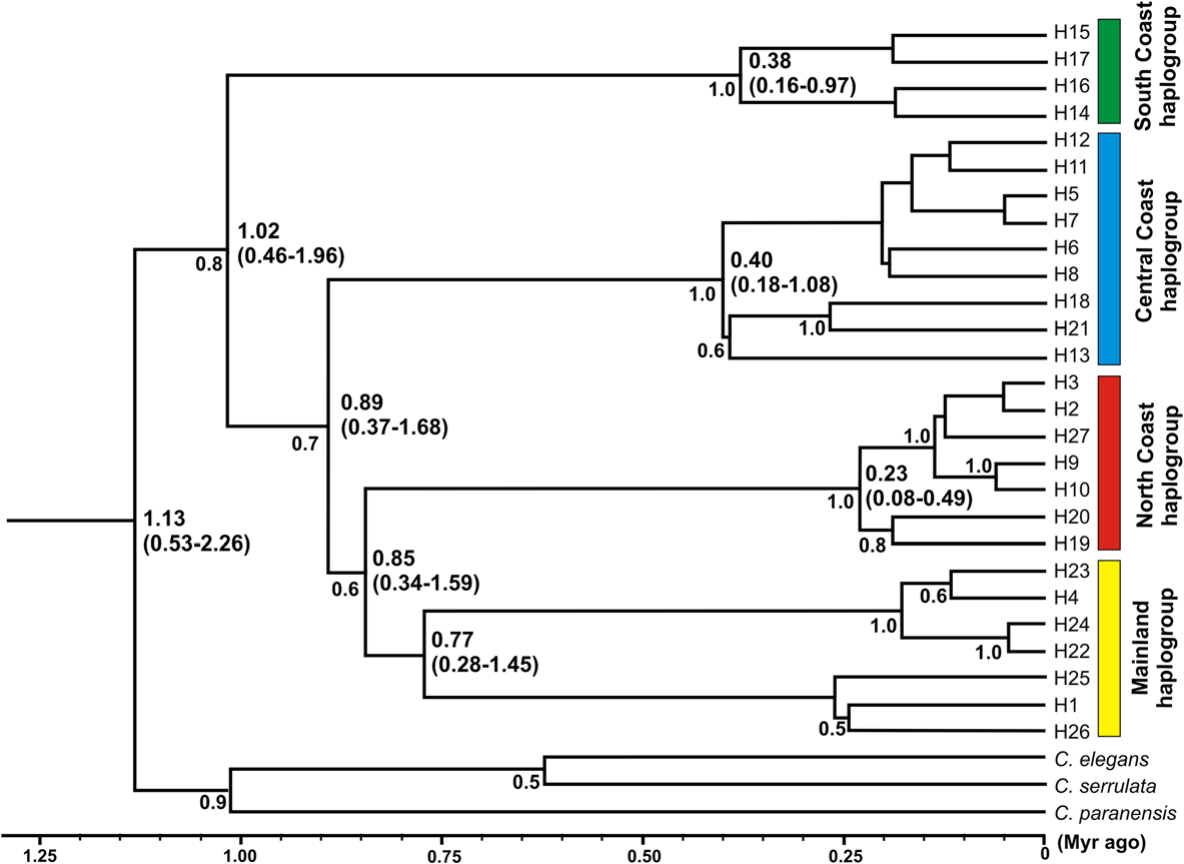
**Bayesian phylogenetic tree.** Bayesian chronogram, with clade posterior probability (>0.5) shown externally to the branches, and point estimates and confidence intervals for the ages (in million of years) are presented next to selected branches.

The haplotype network (Figure 2) resulted in four geographically structured haplogroups that were concordant with the SAMOVA results and are depicted in Figure 3a. The four haplotypes of the southernmost population (P1) formed one haplogroup (South Coast, green). The haplotypes from the other coastal populations formed the haplogroups Central Coast (blue) and North Coast (red), respectively (Table 2). The remaining haplotypes belonging to the populations P11, P12, P13, and P14 were grouped into a Mainland haplogroup (yellow), considering that the last three were collected outside of the SACP. The central haplotype H26, whose grouping may be considered ambiguous in the network, was grouped with its respective clades according to the Bayesian phylogeny (See below; Figure 4). The P6 and P7 populations were the only ones with haplotypes belonging to different haplogroups in the network. The H19 haplotype that belongs to the North Coast haplogroup (red) was also sampled in P6 and the H21 haplotype that belongs to the Central Coast haplogroup (blue) was sampled in P7 (Figure 3a; Table 2). A preeminent feature of the network was that all four clades have a star-like pattern, in which infrequent haplotypes are derived from a common central haplotype with only one or two substitutions. It is also noteworthy that the four haplotypes shared among populations (H14, G1; H5, G2; H2 and H19, G3; H4, G4) were internal to the network haplotypes.

### Phylogenetic relationships and divergence times

The Bayesian analysis (Figure 4) presented the same four major geographically structured clades that were identified in the haplotype network analysis. As expected, given the low sequence divergence in this species, support values for most nodes were moderate to low, especially within the main clades. Haplogroups South, Central and North Coast formed well-supported clades. The estimated divergence time between *C. heterophylla* cpDNA haplotypes and the outgroup was 1.13 (0.53-2.26) Myr ago. The most ancient divergence within *C. heterophylla* was between the South Coast haplogroup and the others at approximately 1 Myr ago. The Central Coast haplogroup separated from the North Coast and Mainland haplogroups approximately 0.89 (0.37-1.68) Myr ago, and the latter two diverged approximately 0.85 (0.34-1.59) Myr ago (Figure 4). The major diversification inside the haplogroups occurred within the last 0.4 Myr. The times to the most recent common ancestors (T_MRCA_) for the four haplogroups were 0.23 (0.08-0.49) (North Coast), 0.38 (0.16-0.97) (Central Coast), 0.40 (0.18-1.08) (South Coast) and 0.77 (0.28-1.45) (Mainland) (see Figure 4).

### Intraspecific demography

**Figure 5:**
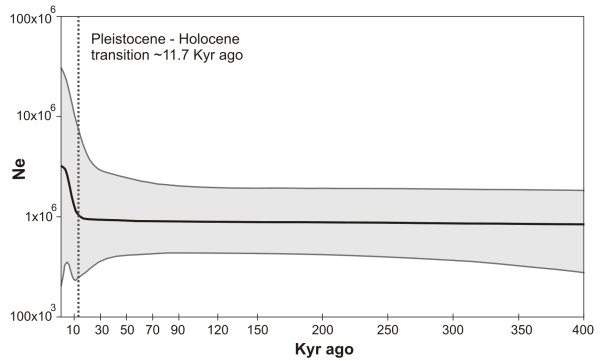
**Bayesian skyline plot.** Bayesian Skyline Plot for *Calibrachoa heterophylla* cpDNA showing the effective fluctuation in population size over time, the thick solid line represents the median estimates and the shaded area the 95% confidence interval. The dashed line indicates the Pleistocene-Holocene transition.

The neutrality tests for each group of populations and for the whole species were mostly non-significant (Table 3). However, Tajima’s *D* and Fu’s *Fs* were negative for all results but significant only for SAMOVA groups G1 and G4 *Fs* (p < 0.02). The Bayesian Skyline Plot for the species (Figure 5) suggested that *C. heterophylla* underwent an increase in effective population size fairly recently, approximately 12 Kyr ago. The very small number of haplotypes, which was most likely due to the sample size, precludes the use of this methodology for each clade separately.

**Table 3:** Summary statistics and population growth rate for the four SAMOVA groups

***SAMOVA group***	***n***	***Populations***	***Tajima's D***	***Fu's Fs***	***h (SD)***	***π (SD)%***
**G1**	33	P1	−1.235	−2.016^§^	0.324 ± 0.098	0.027 ± 0.031
**G2**	92	P2-6	−0.029	−0.371	0.607 ± 0.051	0.157 ± 0.099
**G3**	95	P7-10	−0.614	−1.215	0.647 ± 0.028	0.081 ± 0.061
**G4**	27	P11-14	−1.006	−2.242^§^	0.690 ± 0.079	0.098 ± 0.072
**Total**	**247**		**−0.272**	**−0.415**	**0.879 ± 0.010**	**0.41 ± 0.22**

*π*, nucleotide diversity; *h*, haplotype diversity; SD, standard deviation; *n*, sampling size;.

^§^, significant values (p < 0.02).

## Discussion

### Genetic diversity and population structure

Haplotype diversity in *C. heterophylla* (*h* = 0.879) was similar to that reported for *Petunia axillaris* (*h* = 0.831), *P. altiplana* (*h* = 0.801), *P. bonjardinensis* (*h* = 0.822) and *P. guarapuavensis* (*h* = 0.747), the species that showed the highest haplotype diversity in *Petunia* for the same plastidial intergenic spacers*.* However, the nucleotide diversity for *C. heterophylla* was higher than that of *Petunia* spp. e.g., [7,31]. No other species of *Calibrachoa* has been studied so far, and thus, no comparisons within the genus can be made. The intergenic spacer *trnS-trnG* has proved to be an effective molecular marker for population studies in *Calibrachoa,* showing almost equivalent variability to *trnH-psbA*, in contrast to what was reported for *Solanum* (Solanaceae) [29].

Comparison of the haplotype network, phylogeny and geographical distribution of the haplotypes showed that *C. heterophylla* populations were genetically structured. This was confirmed by the results of AMOVA, in which most of the observed genetic diversity was found among populations. Similar results were also observed in *Petunia exserta* and *P. axillaris*[31] and in seven highland *Petunia* species [7] using the same cpDNA molecular markers. The presence of haplotypes from different haplogroups in P6 and P7 is possibly the result of very recent contact because these populations are geographically adjacent between two distinct geographic clusters (G2 and G3). This highly structured distribution is not surprising when the biology of the species and the molecular markers used are taken into consideration. Chloroplasts are inherited maternally in *Petunia*[37] and *Calibrachoa* species (J.R. Stehmann, Universidade Federal de Minas Gerais, personal communication) and mechanisms of long distance seed dispersal are absent in petunia species [6], which prevents cpDNA gene flow among populations. Studies of plants from coastal environments around the World also showed patterns of geographical structure that may be related to environmental instability mainly due to Quaternary sea level changes: South America [8], Europe [18,19,38], Asia [20], and North America [39].

### Genetics structure *vs* geology and climate

**Figure 6:**
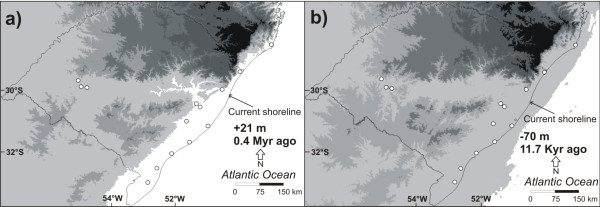
**Sea level changes before and during South Atlantic coastal plain formation.** Approximate paleogeographic reconstruction of sea level, based on reports in the literature and plotted on current data of altitude and bathymetry. The white circles identify the position of the current *Calibrachoa heterophylla* populations studied in this work. **a)** Maximum transgressive event which led to the formation of Barrier–Lagoon System I, Middle Pleistocene, 0.4 Myr ago, 21 m above current sea level [45]. **b)** The Pleistocene-Holocene transition, 11.7 Kyr ago, 70 m below current sea level [51]. The Patos and Mirim Lagoons are not shown because there are no accurate data about their conformation in this period.

The Bayesian chronogram indicated that the diversification that gave rise to the four geographic haplogroups occurred between 1 and 0.85 Myr ago (Figure 4), before the beginning of the contemporary configuration of the SACP (approximately 0.4 Myr ago). In this period, the SACP and the adjacent continental shelf that were exposed during regression periods (Figure 6a) were deeply dissected by paleochannels that carried water from inland basins to the Atlantic ocean, before the formation of the Patos and Mirim Lagoons (Figure 3a; [40]). The paleochannels were interspersed by pre-Cambrian geological formations located in the Central-North region of the SACP (mouth of the Jacuí River) and in the Southern portion (the mouth of the Jaguarão River, located south of the “Southeast Sierra”; Figures 1 and 3a). In the area of the present day distribution of *C. heterophylla*, two main paleochannels (Jacuí and Jaguarão) correlate very well with the distributional limits of the three haplogroups (South, Central and North Coast; Figure 3a). Although there were several transgression/regression events between ~1 and 0.4 Myr ago, the transgressions were less intense than the most recent ones (Figure 4). These data suggest a scenario where the paleochannels may have been the main barriers responsible for the geographically restricted range of the three haplogroups. The presence of riverine barriers has been suggested to play a significant role in delimiting the geographical distribution of species and generating phylogeographic breaks for species of fauna [41-43] and flora [6] on the Brazilian Atlantic Coast. Because few phylogeographic studies have been conducted in the SACP, we report here for the first time the result of rivers acting as barriers to gene flow in a species from this region. This corroborates the proposal [44] of riverine barriers acting as a limiting factor for the geographical distribution of species and contributing to the diversification among populations.

The only sample sites not affected by the Great Marine Transgression that occurred around 0.4 Myr ago were P12-14 (Figure 6a). The inland region (G4) may have acted as a refuge in relation to marine transgressions because the current populations in this region, despite the reduced sampling, showed the highest value of haplotype diversity among SAMOVA groups (Table 3). Moreover, the Bayesian chronogram indicated the Mainland haplogroup was the oldest (0.77 Myr), nearly twice the age of the remaining haplogroups (Figure 4). These results suggest that inland populations may have given rise to the current haplogroups found in the SACP by a colonization route from west to east, which was proposed for flora migration from Andean region to Atlantic Coastal in South America [25]. In some regions the disappearance of geographical barriers may have favored contact among populations that were originally from different lineages, as in the case of populations P6 and P7.

The period of greatest diversification of *C. heterophylla* seems to have occurred in recent times, less than 0.4 Myr ago (Figure 4). A major transgression event occurred around this time where the sea level rose >20 m and most of the lowlands on the coast were submerged [45]. This may have eliminated much of the diversity already present (Figure 6a). However, this and the three subsequent major transgressions formed a series of four sand barriers parallel to the coast that progressively increased the availability of coastal areas after the regressions and may have allowed for the genetic diversity observed today. Several studies have associated genetic diversity and historical biogeography of species distributed in eastern South America with influences of Quaternary climatic fluctuations [46]. The same molecular markers showed that most of the genetic diversity of highlands petunia arose in a similar period [7].

After transgression/regression events, newly formed coastal habitats with the absence of competition provide ecological opportunities for species. These events allow the occurrence of evolutionary processes similar to those occurring on islands and/or mountaintops [47]: rapid diversification driven by ecological opportunities. Similar results demonstrated a rapid diversification of freshwater fishes in the SACP and correlated this with intense environmental changes that were a consequence of Quaternary sea level fluctuations [48]. Additionally, the Quaternary marine transgressions/regressions in the SACP were a major factor in *Deutamys kempi* (a rodent) diversification [49].

### Demography

A similar pattern of population expansion was observed in each of our different analyses (BSP, neutrality tests, network diagram and phylogenetic tree), suggesting a general demographic pattern for the whole species. However, although all neutrality tests were negative (an indication of population expansion), most were not significant. This absence of significance may be explained by being conservative and not very powerful to detect older population size changes [50] compared with BSP. The star-like patterns of clades in the haplotype network and the relatively extensive geographical distribution of the majority of the derived haplotypes throughout coastal areas suggest that rapid colonization occurred in some regions. The recent expansion detected by the BSP (Figure 5) may be a sign of recent expansion linked to the Pleistocene-Holocene transition approximately 12 Kyr ago [9]. At this time, environmental change occurred in the SACP due to marine transgressions/regressions, such as the formation of the Pleistocene Barrier III and Patos and Mirim Lagoons. The sea level was 70 m below what it is now [51], providing a large coastal plain that could be colonized by vegetation (Figure 6b). Moreover, with the beginning of the Holocene, the increase in temperature and humidity may have induced vegetation expansion [11,12]. These environmental changes at the beginning of the Holocene are present in the geologic record from the SACP and adjacent areas in southern Brazil [14]. Quaternary climatic fluctuations were likely strong enough to influence population sizes and, consequently, affect genetic diversification at shallow time scales and community dynamics at smaller scales [52]. Global studies of plant species have also demonstrated population expansions in the same period and have correlated these results with Holocene environmental changes [53,54].

### Main insights and implications for conservation

Despite historical herbarium records, *C. heterophylla* was absent in Uruguay and in some Brazilian locations. This may be because of local extinctions that could have occurred naturally, but more likely, it is because of an anthropic influence, as the entire coastal area has undergone severe human-induced changes in recent decades, such as progressive urbanization, human occupation, development of cattle ranching and agriculture [55]. To preserve the genetic diversity of *C. heterophylla,* specific strategies must be implemented. We propose that populations with higher genetic diversity belonging to each of the four haplogroups must be conservation priorities. Special attention should be given to the South Coast haplogroup represented in only one population (P1) and to P6 and P7 populations, the only ones with haplotypes of distinct haplogroups. The SACP deserves special attention mainly because it has been shaped by the confluence of taxa originating in tropical and temperate regions. These transitional regions are extremely important for the processes of diversification and speciation. The preservation of species that occur in these areas can be used as a model for biological responses to environmental or climatic changes [56].

## Conclusions

The present phylogeographic study of *C. heterophylla* reveals the influence of complex geological and climatic events on patterns of diversification and distribution in this wild petunia species. The fact that the inland populations are grouped in a haplogroup found to be older than those from the coast and that the inland was not noticeably affected by Quaternary sea level changes suggest that *C. heterophylla* originated inland and subsequently colonized the SACP. The major diversification of *C. heterophylla* that occurred less than 0.4 Myr ago is likely to be related to Quaternary sea level oscillations, and earlier variability may have been lost as a consequence of marine transgressions that occurred before the formation of barriers I-IV. Furthermore, paleochannels may have been important for structuring the three haplogroups found. A recent population expansion detected by the BSP seems to be related to an increase in temperature and humidity in the SACP that occurred in the beginning of the Holocene.

The results highlight the substantial contribution of studies of species growing outside of the forests to our knowledge of past vegetation and climate dynamics in the Neotropics. However, more phylogeographic studies of SACP plant species are needed to determine if the phylogeographic history of *C. heterophylla* is representative of multiple taxa or if it is simply an isolated case. If the same or similar patterns were found in other endemic plants, it would suggest that a common mechanism was responsible for driving diversification in this area.

Even single locus phylogenies may not describe exactly the species’ evolutionary history, the cpDNA intergenic spacers *trnG-trnS* and *psbA-trnH* combined are successfully informative to draw the phylogeographic pattern in *Calibrachoa* species [32,33] and related genus *Petunia*[7].

## Methods

### Sample collection, DNA extraction, amplification, and sequencing

Individuals of *C. heterophylla* were intensively sampled over three years across the species’ known distribution, from Laguna (Santa Catarina Brazilian State) to Cape Polonio (Uruguay), including the adjacent northern and southern areas. Despite the existence of herbarium records, the species was not found in Uruguay or in some Brazilian locations. Furthermore, three populations were found out of the SACP in sandy soils along the riverbanks of the inland that were more than 500 Km distant from the South Atlantic Coast (Figure 3a). We sampled a total of 247 individuals from 14 collection sites (Figure 3a) with regular distance intervals, reflecting the distribution of the species (Table 4). Vouchers were deposited in the herbarium (BHCB, Universidade Federal de Minas Gerais, Belo Horizonte, MG, Brazil), and young leaves were carefully collected for genetic analysis. Dried leaves were frozen in liquid nitrogen and ground to a fine powder, and DNA was extracted from the powdered leaves [57].

**Table 4:** **Sampling details of *Calibrachoa heterophylla *populations used in the study**

**Population**	***n***	**Collection site**	**Geographical coordinates**	Voucher*
P1	33	Santa Vitória do Palmar/RS	32° 59' 15''S/52° 43' 56''W	BHCB 104907
P2	26	Rio Grande/RS	32° 31' 26''S/52° 32' 48''W	BHCB 104902
P3	02	Mar Grosso, São José do Norte/RS	32° 02' 47''S/52° 00' 36''W	BHCB 104900
P4	33	Bojuru, São José do Norte/RS	31° 40' 02''S/51° 25' 30''W	BHCB 104880
P5	13	Mostardas/RS	31° 06' 33''S/50° 54' 04''W	BHCB 104895
P6	26	Barba Negra Island, Barra do Ribeiro/RS	30° 28' 31''S/51° 08' 46''W	BHCB 116990
P7	30	Barra do Ribeiro/RS	30° 25' 13''S/51° 13' 30''W	BHCB 116994
P8	24	Santo Antônio da Patrulha/RS	29° 53' 34''S/50° 25' 46''W	BHCB 104866
P9	23	Torres/RS	29° 25' 56''S/49° 47' 53''W	JRS 03251
P10	10	Laguna/SC	28° 27' 36''S/48° 45' 54''W	BHCB 143143
P11	03	Arambaré/RS	30° 55' 09''S/51° 29' 46''W	BHCB 143123
P12	11	Cacequi River, Cacequi/RS	29° 53' 41''S/54° 51' 13''W	BHCB 117016
P13	09	Santa Maria River, Cacequi/RS	29° 51' 17''S/54° 54' 31''W	BHCB 117021
P14	04	São Francisco de Assis/RS	29° 34' 59''S/55° 06' 03''W	BHCB 102097

*BHCB: Herbarium, Department of Botany, Biological Sciences Institute, Universidade Federal de Minas Gerais, Belo Horizonte, Brazil; JRS: João Renato Stehmann. *n,* sampling size.

The *trnH-psbA* and *trnS-trnG* cpDNA intergenic spacers were amplified and sequenced using primers previously described [58,59], respectively. Other plastid (*psbB-psbH, trnL-trnF* and *trnS-trnM* intergenic spacers) and nuclear (ITS and *Leafy* intron 2) loci were tested in representative samples, but no significant variation was found. PCR amplification and sequencing were conducted as described in other petunia species [31]. Sequences were deposited in GenBank (accession numbers: JQ072006-JQ072022, *trnH-psbA,* and JQ082455-JQ082470, *trnS-trnG*).

For all collection localities, no specific permits were required because the land is not privately owned or protected and the field studies did not involve endangered or protected species. This work was conducted under MP 2.186-16 of the Brazilian Federal Government.

### Sequence variation and population genetic structure

The sequences were aligned manually using GeneDoc [60]. Because poly-A/T regions and small inversions were highly variable and homoplastic in preliminary phylogenetic analyses, they were not considered for further analyses, as described previously e.g., [61,62]. The two cpDNA spacers were concatenated in all analyses. Contiguous insertion/deletion events longer than one base pair (bp) were treated as single mutation events [63].

Standard diversity indices including haplotype diversity (*h*), nucleotide diversity (*π*) [64], and analysis of molecular variance (AMOVA [65]) were obtained with Arlequin 3.5.1.2 [66]. The AMOVAs were conducted using 1,000 permutations among collection sites and Φ_ST_ (pairwise differences). Mantel test between genetic and geographical distances and Spatial autocorrelation analyses (1,000 permutations) were performed with the program Alleles in Space 1.0 (AIS [67]). Close sites with small sample sizes were merged.

Historical barriers to gene flow were identified using Spatial Analysis of Molecular Variation in SAMOVA 1.0 [68] with 1,000 permutations. This method identifies geographically homogeneous groups of populations that are maximally differentiated from each other, and it attempts to maximize the proportion of total genetic variation among groups of populations (Φ_CT_).

### Phylogenetic analyses and divergence times

The evolutionary relationships between haplotypes were estimated using the Median-Joining method (ϵ = 0 [69]) implemented in the Network 4.6 program (available at http://www.fluxus-engineering.com). Sequences of *Calibrachoa paranensis* (Dusén) Wijsman*, C. serrulata* (L. B. Sm. & Downs) Stehmann & Semir, and *C. elegans* (Miers) Stehmann & Semir that belong to the same subgenus of *C. heterophylla*[32] were also included in the analysis (Additional file 2).

Phylogeny and divergence times among haplotypes were estimated using a Bayesian approach with BEAST 1.6.1 [70]. Two runs of 10^8^ chains were conducted, sampling every 1,000 generations. The settings used were the Yule tree prior, the HKY substitution model with four gamma categories and the strict molecular clock. The substitution rate used was 2.8 × 10^-9^ (± 5.4 × 10^-11^) substitutions per site per year, or 0.56% per million years. This rate was chosen because it was previously estimated for *Petunia* for the same loci studied here [7] and it is similar to those found in other plant groups (0.22–0.58% per Myr [71]; 0.26–0.92% per Myr [72]). The best-fit model of sequence evolution was identified in Modeltest 3.7 [73] using the Akaike Information Criterion [74]. Tracer 1.5 (available at http://beast.bio.ed.ac.uk/Tracer) was used to check for convergence of the Markov chains and adequate effective sample sizes (>200) after the first 2 × 10^7^ chains were deleted as “burn-in”. A maximum clade credibility tree was obtained using TreeAnnotator, part of the BEAST software package. Statistical support was determined by assessing the Bayesian posterior probabilities.

### Demographic analyses

The demographic history of *Calibrachoa heterophylla* was investigated using several methods, such as Tajima’s *D*[75] and Fu’s *Fs*[76] neutrality tests calculated with Arlequin for the entire dataset and for each haplogroup (found in the network and phylogeny, see below). In addition, the Bayesian Skyline Plot (BSP [70]; implemented in BEAST and Tracer) was used to estimate the dynamics of changes in population size over time for the *C. heterophylla* dataset. The settings for the substitution model and substitution rate were the same as those used for the phylogenetic analysis.

## Availability of supporting data

Sequences are deposited in GenBank (accession numbers: JQ072006-JQ072022, *trnH-psbA,* and JQ082455-JQ082470, *trnS-trnG*) and in Dryad repository: http://dx.doi.org/10.5061/dryad.564ms. The concatenated alignment is available in http://dx.doi.org/10.5061/dryad.10v90.

## Competing interests

The authors declare that they have no competing interests.

## Authors’ contribution

LBF and GM planned, designed, and led the project; GM did the laboratory work; GM, APLL, JNF and SLB ran the analyses; GM and JNF collected the plant material; GM, SLB and LBF wrote most of the text. All authors have contributed in the preparation of the study and commented on and approved the final manuscript.

## Authors’ information

Collectively the group is interested in investigating evolutionary processes, plant speciation, conservation genetics, phylogeographic patterns and molecular systematics.

## Supplementary Material

Additional file 1: Table S2 Haplotypes identified in the cpDNA of *Calibrachoa heterophylla.* Click here for file

Additional file 2: Table S1 Information about outgroup used in the phylogenetic analysis. Click here for file
