# Supplementary material for: Geological and climatic changes in quaternary shaped the evolutionary history of Calibrachoa heterophylla, an endemic South-Atlantic species of petunia
Source: BMC Evol Biol. 2013 Aug 29;13:178. doi: 10.1186/1471-2148-13-178 (PMC3765879; doi:10.1186/1471-2148-13-178)
Supplement: Additional file 1: Table S2 — Haplotypes identified in the cpDNA of Calibrachoa heterophylla. [file 1471-2148-13-178-S1.doc]

**Table S2 -** Haplotypes identified in the cpDNA of *Calibrachoa heterophylla*.

* 5-bp duplication (ATTTT); *n*, number of individuals with each haplotype.

| **Nucleotide position** | | | | | | | | | | | | | | | | | | | | | | | | | | | | | | | | | | | | | | |
| --- | --- | --- | --- | --- | --- | --- | --- | --- | --- | --- | --- | --- | --- | --- | --- | --- | --- | --- | --- | --- | --- | --- | --- | --- | --- | --- | --- | --- | --- | --- | --- | --- | --- | --- | --- | --- | --- | --- |
|  | ***trnH-psbA*** | | | | | | | | | | | | | | | | |  | | ***trnS-trnG*** | | | | | | | | | | | | | | | |  |  | **Population**  **sampled** |
| **Haplotype** | 26 | 27 | 46 | 75 | 116 | 128 | 171 | 192 | 205 | 243 | 255 | 275 | 280 | 314 | 353 | 443 |  | | 706 | | 718 | 724 | 741 | 749 | 756 | 781 | 788 | 811 | 847 | 864 | 900 | 908 | 983 | 1028 | 1224 |  | ***n*** |
| H1 | T | A | A | T | G | G | T | C | T | A | A | G | T | G | C | A |  | | T | | G | G | T | A | T | G | C | T | A | A | G | * | G | C | C |  | 3 | P11 |
| H2 | . | . | . | . | . | . | . | . | . | . | . | . | G | . | . | . |  | | . | | . | A | . | C | . | . | . | . | . | . | . | * | T | . | . |  | 42 | P9-8 |
| H3 | . | . | . | . | . | . | . | . | . | . | . | . | G | . | . | . |  | | . | | . | A | . | C | . | . | . | G | . | . | . | * | T | . | . |  | 1 | P9 |
| H4 | . | . | . | . | . | . | . | . | . | . | . | C | . | . | . | . |  | | . | | . | . | . | . | . | . | . | . | . | . | . | * | T | . | . |  | 14 | P12-14 |
| H5 | . | . | . | . | . | . | . | A | . | . | . | . | . | . | . | . |  | | C | | . | . | . | . | . | . | T | . | . | . | . | - | T | . | . |  | 55 | P2-5 |
| H6 | . | . | . | . | . | . | . | A | . | . | . | . | . | . | . | . |  | | C | | A | . | . | . | . | . | T | . | . | . | . | - | T | . | . |  | 1 | P5 |
| H7 | . | . | . | . | . | . | . | A | . | . | . | . | . | . | . | . |  | | C | | . | . | . | . | . | . | T | . | . | . | . | - | T | . | G |  | 2 | P2 |
| H8 | . | . | . | . | . | . | . | A | . | . | . | . | . | A | . | . |  | | C | | . | . | . | . | . | . | T | . | . | . | . | - | T | . | . |  | 10 | P2 |
| H9 | . | . | . | . | . | . | . | . | . | . | . | . | G | . | . | . |  | | . | | . | A | . | C | . | . | . | . | . | . | T | * | T | . | . |  | 3 | P8 |
| H10 | . | T | . | . | . | . | . | . | . | .. | . | . | G | . | . | . |  | | . | | . | A | . | C | . | . | . | . | . | . | T | * | T | . | . |  | 1 | P8 |
| H11 | . | T | . | . | . | . | . | A | . | . | . | . | . | . | . | . |  | | C | | . | . | . | . | . | . | T | . | . | . | . | - | T | . | . |  | 1 | P4 |
| H12 | . | . | . | . | . | . | . | A | . | . | . | . | . | . | . | . |  | | C | | . | . | . | . | . | A | T | . | . | . | . | - | T | . | . |  | 4 | P4 |
| H13 | . | . | . | . | . | . | . | A | . | . | . | . | . | . | . | . |  | | . | | . | . | . | . | . | . | . | . | . | . | . | - | T | . | . |  | 1 | P4 |
| H14 | C | . | . | . | . | T | . | . | . | . | . | . | . | . | T | . |  | | . | | . | . | . | . | . | . | . | . | . | T | . | * | T | . | . |  | 27 | P1 |
| H15 | C | . | . | . | . | T | . | . | . | . | . | . | . | . | T | T |  | | . | | . | . | . | . | . | . | . | . | . | T | . | * | T | . | . |  | 4 | P1 |
| H16 | C | . | . | . | . | T | . | . | . | . | . | . | . | . | T | . |  | | . | | A | . | . | . | . | . | . | . | . | T | . | * | T | . | . |  | 1 | P1 |
| H17 | C | . | . | . | . | T | . | . | . | . | G | . | . | . | T | . |  | | . | | . | . | . | . | . | . | . | . | . | T | . | * | T | . | . |  | 1 | P1 |
| H18 | . | . | T | . | . | . | . | A | . | . | . | . | . | . | . | . |  | | . | | . | . | . | . | . | . | . | . | T | . | . | - | T | G | . |  | 15 | P6 |
| H19 | . | . | . | . | . | . | . | . | . | . | . | . | . | . | . | . |  | | . | | . | A | . | C | . | . | . | . | . | . | . | * | T | . | . |  | 37 | P6-7 |
| H20 | . | . | . | . | . | . | . | . | G | . | . | . | . | . | . | . |  | | . | | . | A | . | C | . | . | . | . | . | . | . | * | T | . | . |  | 1 | P7 |
| H21 | . | . | T | . | . | . | . | A | . | . | . | . | . | . | . | . |  | | . | | . | . | . | . | . | . | . | . | . | . | . | - | T | G | . |  | 3 | P7 |
| H22 | . | . | . | . | . | C | . | . | . | . | . | C | . | . | . | . |  | | . | | . | . | . | . | . | . | . | . | . | . | . | * | T | . | . |  | 6 | P12 |
| H23 | . | . | . | A | . | . | . | . | . | . | . | C | . | . | . | . |  | | . | | . | . | . | . | . | . | . | . | . | . | . | * | T | . | . |  | 1 | P12 |
| H24 | . | . | . | . | . | C | . | . | . | C | . | C | . | . | . | . |  | | . | | . | . | . | . | . | . | . | . | . | . | . | * | T | . | . |  | 1 | P12 |
| H25 | . | . | . | . | T | . | . | . | . | . | . | . | . | . | . | . |  | | . | | . | . | . | . | G | . | . | . | . | . | . | * | T | . | . |  | 1 | P13 |
| H26 | . | . | . | . | . | . | . | . | . | . | . | . | . | . | . | . |  | | . | | . | . | . | . | . | . | . | . | . | . | . | * | T | . | . |  | 1 | P13 |
| H27 | . | . | . | . | . | . | A | . | . | . | . | . | G | . | . | . |  | | . | | . | A | A | C | . | . | . | . | . | . | . | * | T | . | . |  | 10 | P10 |
