# Supplementary material for: Geological and climatic changes in quaternary shaped the evolutionary history of Calibrachoa heterophylla, an endemic South-Atlantic species of petunia
Source: BMC Evol Biol. 2013 Aug 29;13:178. doi: 10.1186/1471-2148-13-178 (PMC3765879; doi:10.1186/1471-2148-13-178)
Supplement: Additional file 2: Table S1 — Information about outgroup used in the phylogenetic analysis. [file 1471-2148-13-178-S2.doc]

**Table S1 – Information about outgroup used in the phylogenetic analysis.**

| ***Species*** | ***Geographical Coordinates*** | ***Collection number*** | ***Genbank accession*** |
| --- | --- | --- | --- |
| *Calibrachoa paranensis* (Dúsen) Wijsman | 25° 27' 57''S/ 49° 43' 16''W | JRS 4215 | JQ082472; JQ082475 |
| *Calibrachoa serrulata* (L.B.Sm. & Downs) Stehmann & Semir | 28° 23' 16''S/ 49° 32' 38''W | JRS 4394 | JQ082473; JQ082476 |
| *Calibrachoa elegans* (Miers) Stehmann & Semir | 20º 05' 35"S/ 43º 59' 01"W | JRS 2414 | JQ082471; JQ082474 |
